# PyCorrFit—generic data evaluation for fluorescence correlation spectroscopy

**DOI:** 10.1093/bioinformatics/btu328

**Published:** 2014-05-13

**Authors:** Paul Müller, Petra Schwille, Thomas Weidemann

**Affiliations:** ^1^Biotechnology Center, Technische Universität Dresden, 01307 Dresden and ^2^Max Planck Institute of Biochemistry, Cellular and Molecular Biophysics, 82152 Martinsried, Germany

## Abstract

**Summary:** We present a graphical user interface (PyCorrFit) for the fitting of theoretical model functions to experimental data obtained by fluorescence correlation spectroscopy (FCS). The program supports many data file formats and features a set of tools specialized in FCS data evaluation.

**Availability and implementation:** The Python source code is freely available for download from the PyCorrFit web page at http://pycorrfit.craban.de. We offer binaries for Ubuntu Linux, Mac OS X and Microsoft Windows.

**Contact:** paul.mueller@biotec.tu-dresden.de and weidemann@biochem.mpg.de

## 1 INTRODUCTION

Fluorescence imaging applications provide a wealth of information about the spatiotemporal distribution of fluorescent molecules in tissues or cells. However, time-lapsed imaging and related protocols, e.g. fluorescence recovery after photobleaching (FRAP), operate with a temporal resolution of sub-seconds, which is not fast enough to access free diffusion of mid-sized proteins. About a decade ago it was demonstrated that a confocal imaging setup can be extended by a fluorescence correlation spectroscopy (FCS) unit (Wachsmuth *et al.*, 2003). Such a fluorescence fluctuation microscope can be used to measure binding and diffusion at specific positions with a sub-microsecond resolution (Kim *et al.*, 2007). Since then, most of the major microscope companies developed FCS extensions for their products, and fluorescence fluctuation analysis became an integral part of the analytical repertoire in biomedical research.

**Fig. 1.**
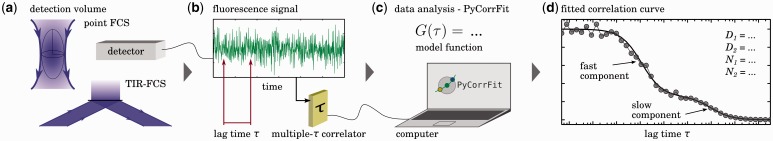
FCS from data acquisition to parameter extraction. (**a**) Confocal point FCS (top) and total internal reflection (TIR-) FCS (bottom) arrangements generate different implementations of a femtoliter-sized detection volume. (**b**) The fluctuating fluorescence signal (e.g. from diffusing particles) is recorded and a multiple-τ algorithm is applied using a software or hardware correlator. (**c**) The obtained correlation curve is processed using PyCorrFit. A theoretical model function *G*(τ) is fitted to the correlation curve to extract physical parameters. (**d**) Exemplary fit (black) to a measured correlation curve (gray) yielding a slow and a fast diffusing species of particles at different concentrations. Note that the correlation curve is calculated and displayed on a logarithmic scale of the lag time τ

FCS is based on the temporal correlation analysis of a fluctuating fluorescence signal (Figure 1). Using microscopes with multiple detection channels, a measurement generates a set of auto- and cross-correlation curves, which contains information about dynamic molecular processes associated with the fluorescence emission from a femtoliter-sized detection volume (Schwille, 2001). Correlation curves are interpreted by means of theoretical model functions describing the underlying physical processes. For example, diffusion-driven fluctuations must be evaluated for a particular geometry of the detection volume. In addition, molecular behavior like reversible binding at sites within the detection volume, the population of photophysical dark states due to triplet or blinking phenomena and photobleaching simultaneously affect the measured shape of the correlation curves and must therefore be theoretically treated. In practice, experimental correlation data are evaluated by fitting appropriate model functions, thereby extracting parameters that describe the spatial and dynamical properties of the system.

Custom-made evaluation scripts for a particular FCS instrument are usually designed for its specific optics (confocal, total internal reflection, etc., Figure 1a) and often contain only a limited set of model functions. The underlying rationale for defining parameters and models may not be well-described, leaving the user with uncertainties for interpretation. Furthermore, the revision and parameter tweaking of correlation data from different sources can be a time-consuming procedure, raising the demand for a framework that is capable of dealing with any conceivable scenario in which correlation curves need to be processed. PyCorrFit offers such a framework, focusing on four major notions: (i) the use of established fitting algorithms; (ii) the possibility to implement customized model functions and data file formats; (iii) a comprehensive annotation of the evaluation dataset; and (iv) a user-friendly environment.

## 2 IMPLEMENTATION

Traditionally, the signal intensity fluctuations are computed with a multiple-τ algorithm as presented by Schätzel (1990). This algorithm produces correlation curves on a quasi-logarithmic time scale of the lag time τ, thereby maintaining temporal resolution across orders of magnitude while keeping the file size manageable.

Correlation curves, when present in a supported file format [e.g. ‘.fcs’ (Zeiss), ‘.sin’ (correlator.com) and ‘.ASC’ (ALV)], can directly be imported. Additional data formats can be added as separate modular files to the library of PyCorrFit.

The evaluation of experimental correlation curves involves the non-linear least squares fit of a model function. Among others, we use the conventional Levenberg–Marquardt algorithm (Levenberg, 1944) as implemented in the *leastsq* function of Python’s *SciPy* package.

PyCorrFit offers weighted fitting, i.e. the use of variances to weight the effect of single data points (τ-channels) on the overall fit. For single curves, the weights can be calculated from the variance of the difference between the experimental correlation data, and either the model function or a spline fit to the experimental data. When fitting multiple correlation curves measured for the same molecular system, the local variances of the individual curves can also be used as weights for the curve average. After fitting, the difference between the experimental correlation curve and the model function (residuals) are displayed to identify systematic deviations along the τ-axis, which in turn indicate a wrong parameter set or model.

PyCorrFit comes with a set of pre-implemented model functions covering confocal FCS and total internal reflection FCS (TIR-FCS). These functions include multicomponent diffusion models, triplet dynamics and blinking. To account for special conditions, external user-defined model functions can be imported from a self-written text file (‘.txt’) that follows a simple syntax and may include various mathematical expressions, including the Faddeeva function, which is necessary to describe the evanescent wave component in TIR-FCS. Examples for external model functions treating circular scanning FCS or a combination of diffusion and flow are available at the PyCorrFit web page.

To simplify data analysis, we implemented a number of features that allow to simultaneously operate on larger datasets. This includes a global correction for non-correlated background, batch processing when fitting multiple correlation curves measured in the same system and shared parameters across different fit models, for example, fluorescence cross-correlation spectroscopy (FCCS) experiments (Ries *et al.*, 2010; Weidemann *et al.*, 2002). In addition, the user can visualize parameters by simulating the corresponding correlation functions, which may help in deciding on different models.

PyCorrFit allows for exporting acquired data in various ways: individual correlation curves and fit-functions can be tabulated as comma-separated values (‘.csv’). These files include a header about specifics of the fit, for example, the type of weights that were used. For quick monitoring and reporting, plotted data can be exported as bitmap or vector scaled graphics. Latex formatting is also supported. Furthermore, PyCorrFit allows for saving and restoring the entire session. This is in particular useful when some parameters evolve during the course of the project, and one is situated in revisiting previously evaluated data with a slightly modified fitting procedure.

## 3 CONCLUSION

PyCorrFit is an open-source graphical user interface (GUI) that offers a standardized environment for the analyses of correlation data. Using the program does not require prior knowledge in programming.

While the simple GUI permits a transparent workflow, software developers may supplement its capabilities by adding novel tools, implementing new file formats or manipulating the code for other applications involving a quantitative correlation analysis.

For example, we recently generated a software tool (PyScanFCS) to extract correlation curves from kymographs, the primary data in perpendicular line scanning FCS, and used PyCorrfit for the final evaluation of the correlation functions (Müller *et al.*, 2014).

Funding: This work was gratefully supported by Deutsche Forschungsgemeinschaft (DFG) priority program SFB/TRR 64 “Funktionelle Biomaterialien zur Steuerung von Heilungsprozessen in Knochen- und Hautgewebe – vom Material zur Klinik”.

*Conflict of interest*: none declared.
